# Novel Method of Remotely Monitoring the Face-Device Distance and Face Illuminance Using Mobile Devices: A Pilot Study

**DOI:** 10.1155/2019/1946073

**Published:** 2019-06-11

**Authors:** Rosa María Salmerón-Campillo, Mateusz Jaskulski, Sergio Lara-Cánovas, José Manuel González-Méijome, Norberto López-Gil

**Affiliations:** ^1^Grupo de Ciencias de La Visión (CiViUM), Facultad de Óptica y Optometría, Universidad de Murcia, Murcia, Spain; ^2^Clinical Optics Research Lab (CORL), Indiana University School of Optometry, Bloomington, IN, USA; ^3^Clinical & Experimental Optometry Research Lab (CEORlab)—Center of Physics, University of Minho, Braga, Portugal

## Abstract

Specially developed software (app) was written for handheld electronic devices that uses the device camera and light detector for real-time monitoring of near-work distance and environmental lighting. A pilot study of this novel app employed children using tablet computers in a classroom. Measurements of face-device distance and face illuminance were obtained from two schools where tablets were used regularly. Children were divided randomly into a control group (CG) and intervention group (IG). The app was calibrated in a lab and configured to store average values every 20 seconds in a remote database. In both groups, the app recorded data only when a child's face was present in the camera image. The app darkened the screen for the IG when the face-device distance was shorter than 40 cm. The total mean face-device distance was 36.8 ± 5.7 cm in CG and 47.2 ± 6.5 cm in IG. Children in IG had to accommodate approximately 0.6 D less when using their devices. The mean classroom face illuminance was 980 ± 350 lux in School #1 and 750 ± 400 lux in School #2. The novel method of remotely monitoring and controlling the face-device distance and illuminance can potentially open new paths for myopia prevention and myopia control.

## 1. Introduction

Myopia is one of the principal causes of vision loss worldwide, and its prevalence is increasing [1, 2]. It has become a major public health concern due to comorbidities that can potentially result in blindness [3]. There is an increasing urgency in identifying factors with the highest impact on myopia onset and progression, as well as interventions that could prevent its onset or slow its progression.

Numerous studies show that myopia can be a consequence of an interaction of genetic [4, 5] and environmental factors [6] such as near work [7] and environmental light levels [8, 9]. At the same time, the almost universal use of personal electronic devices in recent years has increased the amount of daily near-work activities, as screens are often viewed closer than printed text [10, 11]. It has been hypothesized that intensive use of electronic devices by young children might trigger the onset and accelerate the progression of myopia [12], and yet children start using them at increasingly young age [13]. In the USA, 2015, the average time spent with mobile devices by children 8 and under was 2.3 h, a threefold increase from 2013, while 38% of the children under the age of 2 have used a mobile device [13].

A meta-analysis published in 2015 involving over 25 thousand subjects between 6 and 18 years of age found a strong correlation between near work and myopia [14]. The association between near work and odds of myopia increased by 2% per each additional diopter-hour of near-work activity. Another meta-analysis evaluating the impact of outdoor activities on the odds of myopia onset indicated a 2% decrease per each additional hour of time spent outdoors per week [15]. These results have been confirmed by a more recent work by Xiong et al. who reported that time spent by children engaged in outdoor activities in high-illumination conditions had a protective effect on myopia onset but not myopia progression [16]. They found a reduction between 2% and 5% in the odds of myopia onset due to an increase of outdoor activity.

In a 2018 study, Wu et al. [17] concluded that high environmental light levels can aid the emmetropization process. It is estimated that the illumination level on a sunny day can be approximately 100,000 lux, while indoors it is typically between 100 and 500 lux [18]. Several schools in Taiwan have implemented more outdoor activities so that children could rest from near-work indoors, and after a one-year study, it was found that the myopia incidence in these children dropped to 8.4% compared to 17.6% in children who did not participate in the study [19].

Performing near work indoors and performing activities outdoors are intertwined parts of everyday lives, and the extent of their separate influence on myopia onset and progression is still not well known due to the fact that it is very difficult to measure these factors in real life conditions and they are inherently correlated with each other (negative correlation). At the same time, if behavioral interventions to address myopia (such as modifying the visual demand during near work, increasing the time outdoors, or increasing classroom illumination) are to be implemented, it is critical to quantify the “dose” and ensure subject compliance with such interventions. This urgently calls for tools and methods that can measure and monitor these factors independently and accurately in real-time.

Unfortunately, until recently, methods were limited to questionnaire responses obtained from children or their parents [20–22]. Example questions include: “how many pages did the child read last week?” or “how many hours did the child spend outside?” Results obtained in this way inherently suffer from poor accuracy and precision because they depend on human memory and biases. As an example, Li et al. found that the correlation between two subsequent surveys related to outdoor activity conducted in an interval of three weeks was just 0.63 and Cronbach's *α* coefficient was an unacceptable 0.61 [23]. Although parents can estimate time spent outside, they are unable to quantify their children's near-work viewing distances and room illumination.

Most recently, modern range-finding technologies have been implemented as wearable clip-on devices that can attach to spectacle frames, which can measure the distance between the device and diffusely reflective objects (such as books) [24]. Other examples include a wearable light-sensing device in the form of a wristband [25] and novel methods to measure the time spent outdoors using ultraviolet exposure biomarkers [26, 27]. A common disadvantage of all of these methods is the fact that they require children to consistently wear unfamiliar pieces of technology.

On the other hand, modern mobile devices, which children already use extensively [12, 13], come equipped with a light sensor, front-facing camera, wireless connectivity, and processing power, which can be used to accurately measure both the face-device distance and face illuminance from the image of the face captured by the camera. Therefore, while being used normally and potentially contributing to myopia development, these devices can automatically monitor myopia-related behaviors, and, if desired, perform interventions, such as showing warnings on the electronic screen.

This study evaluated a novel method of real-time monitoring of near-work distance and face illuminance of children using mobile devices that were equipped with software developed for this purpose.

## 2. Materials and Methods

### 2.1. Overview and Principle of Operation of the App

**Figure 1:**
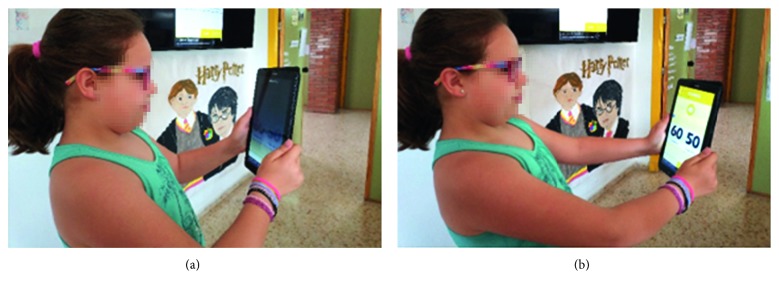
When the student's face-device distance is shorter than a preconfigured distance (in this case, 40 cm), the screen is partially darkened (a), making the student move the device further away (b) to restore normal screen brightness.

Software was loaded onto each tablet as a custom app (not available in the market at the time), which once activated is designed to run continuously in the background of the operating system. This software and the device hardware are together capable of measuring the face-device distance and face illuminance in real-time during normal use of the device. User-determined options can activate “warnings” when certain device-user characteristics were evaluated by the app exceeded user-defined parameters such as a minimum viewing distance. For example, the screen could be darkened when the measured distance was shorter than a certain preselected minimum distance (Figure 1).

The face-device distance measurement algorithm required an individual one-time (per subject and device) calibration procedure, the principle of which is expressed in

(1) $$\begin{array}{c} K=d_{\text{c}}\cdot n_{\text{c}}, \end{array}$$

where *K* is a constant value which depends on the device and can be calculated by equation (1) knowing the value of *d*_c_, which is the calibration distance, and the value of *n*_c_, the number of pixels in the image of the user's head captured by a front camera of the device during the calibration.

The face-device distance *d*_t_ could then be estimated in real-time using the following equation:

(2) $$\begin{array}{c} d_{t}=\frac{K}{n_{t}}, \end{array}$$

where *n*_*t*_ is the number of pixels in the image of the user's head captured by a front camera of the device at time *t*. The methodology is further described in detail in the application patent from López Gil and Liu [28].

Additionally, the app was capable of measuring the face illuminance using two methods: with a built-in, wide field-of-view ambient light sensor (typically situated close to the front camera) [29] and with the front camera itself—by using the pixels in the image of a user's head. In the present study, the former method was used.

The app was configured to store average values of distance (in mm) and illuminance (in lux) along with a timestamp every 20 seconds in a remote database. The app recorded data only when the child's face was present in the image from the front camera with a frame rate of 30 fps.

### 2.2. Calibration of Face-Device Distance Measurements

**Figure 2:**
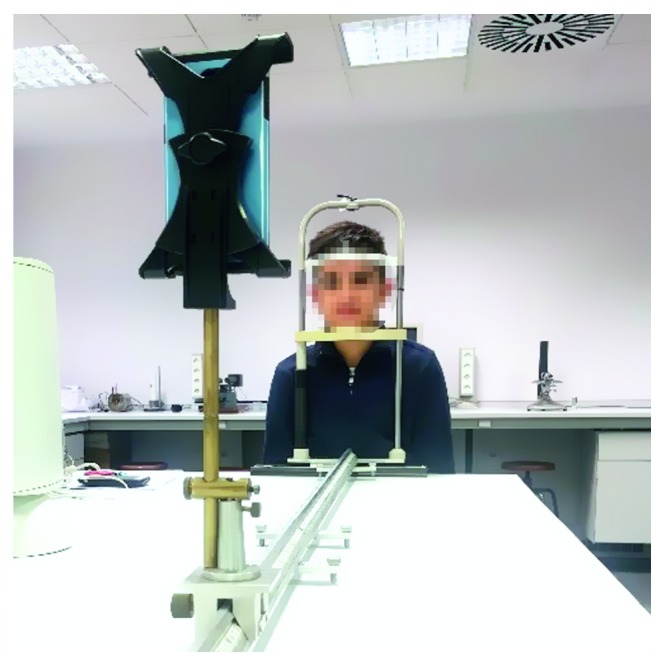
Laboratory setup used for testing the accuracy of face-device distance measurements. Face position was stabilized with a chin and forehead rest, and the mobile device position was adjusted along the optical bench.

The accuracy of distance and illuminance measurements was evaluated in laboratory conditions using the same model device that was to be used in schools (Samsung Galaxy™ Tab A SM-P580 with a 10-inch screen). Repeat measurements were collected from one subject, who used a chinrest to stabilize the position of the head. An optical bench was placed in front of the chinrest, allowing for accurate positioning of the device (±1 mm) between 40 and 250 cm (Figure 2). The minimum distance was limited by the FOV of the camera (46° for a 28 mm equivalent focal length lens). The 2 megapixel images (1920 × 1080 pixel) were sufficient to assess face image size at distances up to 250 cm.

**Figure 3:**
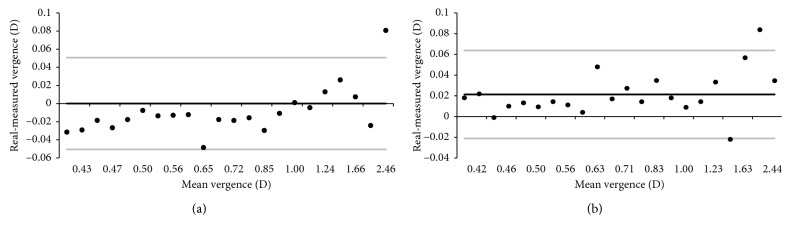
Bland-Altman plots of differences between observed and actual face-device vergence after calibration at 60 cm (a) and 200 cm (b). Black line represents the mean value and grey lines the 5^th^ and 95^th^ percentiles of the difference distributions.

The face-distance measurement calibration was carried out using two different calibration distances (*d*_c_ = 60 cm and 200 cm) to verify how it affected the measurement accuracy. At each calibration distance, the software detected the face, and the operator entered the distance and the software calculated *K* (equation (1)). Subsequently, the position of the device in the optical bench was changed in 10 cm increments, three measurements of face-device distance were recorded by the app, and the average and standard deviation were calculated.  Figure 3 shows the difference between the device-determined vergence (inverse of distance in meters) and the actual face-device vergence. A positive sign for the vergence convention has been used for real stimulus.

The distance measurement error did not exceed 5 mm (equivalent to a vergence error <0.03 D at a viewing distance of 40 cm). These results are similar to those previously reported using the same methodology in other devices [30]. The mean and 95% limit (±1.96 ∗ SD) of the agreement in the vergence measurement (Figure 3(b)) were −0.01 ± 0.05 D and 0.02 ± 0.04 D for the 60 cm and 200 cm calibration, respectively.

### 2.3. Calibration of Face Illuminance Measurements

**Figure 4:**
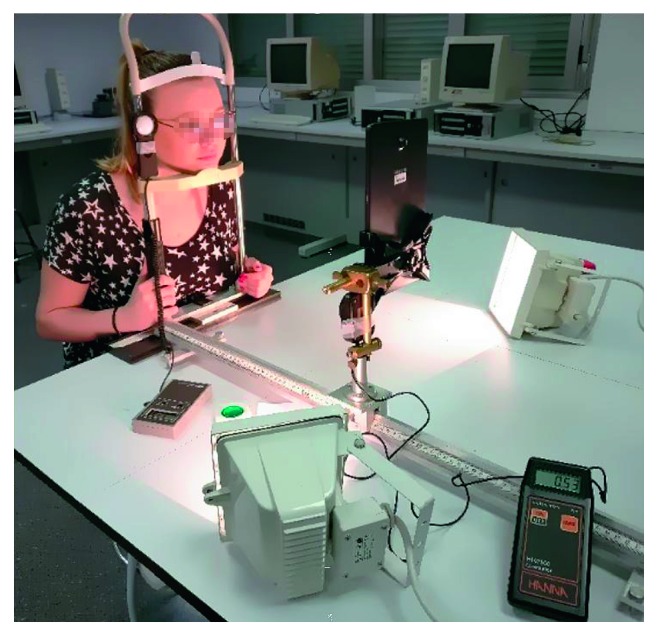
Laboratory setup for evaluating accuracy of illumination measurements. Face position was fixed with a chin and forehead rest. A lux meter was placed temporal to the right eye and in the eye plane.

Calibration of illumination measurement (Figure 4) used a chinrest and optical bench with the face-device distance fixed at 30 cm. A dome-type lux meter (Hanna HI 97500) was situated at the eye level (temporal to right eye). The room was illuminated with two variable-power 500 W incandescent lights placed on the table top at a distance of approximately 40 cm from the face, and the room's ceiling fluorescent lights were either on or off.

**Figure 5:**
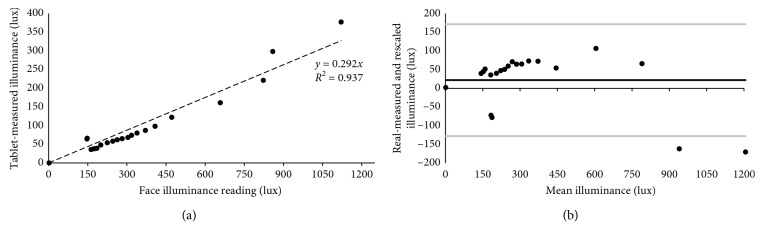
Illuminance measurements as a function of light meter readings. The dotted line in (a) shows the fitting straight line with a slope of 0.292. Bland-Altman plot in (b) shows the same measurement results but rescaled after using the slope value. Black line in (b) represents the mean value and grey lines the 5^th^ and 95^th^ percentiles of the difference distributions.

In total, 21 single measurements of illumination were taken within the range from 10 to 1200 lux (Figure 5). The standard deviation of repeat measures of face illumination obtained by the tablet with the app was corresponded to the instrument error: ±1 lux.

The slope of 0.292 in the linear fit to the data in Figure 5(a) indicates that, in order to obtain real face illumination values, the tablet lux meter readings needed to be multiplied by its inverse (3.425). The lower tablet lux meter [29] readings reflect its wide angle of integration (typically ∼60 degrees). The illuminated face subtended only a portion of this angle, and the wall of the lab, being further away, had lower illuminance from the halogen lights (inverse square law); hence, the solid angle integration (approximately 1.05 sr) resulted in the lower average illuminance. Future developments which measure light only in the pixels in the image of the face taken with the front camera will eliminate the need for this scaling factor.

The four out-lying points seen in Figure 5(a) appear to be aligned together. This occurred because the fluorescent ceiling lights were on for these measurements and the wide angle of integration of the tablet light sensor included light coming from the fluorescent lights on the ceiling in addition to the light reflected from the face. When the four points are not taken into account the fitting parameter, *R*^2^, increases to 0.986. These four out-lying points also increase the interval of confidence in the Bland-Altman plots in Figure 5(b). In order to improve the accuracy of the calibration, the lab conditions should ideally reflect classroom conditions, which are not standardized and can vary significantly between schools.

The mean and 95% limit (±1.96 *∗* SD) of the agreement in the rescaled illuminance measurement (Figure 5(b)) were 21 ± 150 lux.

Face illuminance yielded by light emitted by the screen of a device is usually much lower than room illumination. For instance, illuminance of 190 lux was obtained when the room lights were on and the screen was off, which increased only by 8 lux when the screen was turned on, displaying a white target at full brightness. This result emphasizes that the measured illuminance is reflective of general environmental lighting (e.g., room or sky).

### 2.4. Measurements in Schools

Distance and illuminance measurements were obtained from two schools where tablets were used as a part of their regular teaching programme: CEIP Torrealta in Molina de Segura (School #1) and CEIP Esparragal in Puerto Lumbreras (School #2), both located in the region of Murcia (Spain). The number of participants was 11 and 34 in School #1 and School #2, respectively. Ages ranged from 10 to 13 years (10.6 ± 0.5 years in School #1 and 11.1 ± 0.7 years in School #2). No subjects suffered from any vision problems which would impair their ability to use a tablet at a distance greater than 40 cm. The participation in the study was voluntary. Before the commencement of the study, it was approved by the Ethics Committee of the University of Murcia and the participants were informed about their rights and their parents or legal guardians received an informed consent form in accordance with the guidelines of the Ethics Committee. The research followed the tenets of the Declaration of Helsinki.

The app was installed in 45 tablets (Samsung Galaxy™ Tab A SM-P580), used daily during classes in both schools. Each tablet was associated with one child, and the near-work distance measurement calibration was performed individually to ensure the accuracy of measurements. The room illuminance in both classes was measured using a lux meter (Hanna HI 97500) at three different places, and the range of illuminance was similar to the one used during the accuracy testing.

The children wore their habitual refractive correction and were randomly divided into two groups: control group (CG) with 21 subjects and intervention group (IG) with 24 subjects. The intervention group experienced the partial darkening effect applied to the screen of the device whenever the face-device distance measured by the app was shorter than the preconfigured distance of 40 cm. Children could still interact with the devices but had to move it beyond the preconfigured distance for the screen brightness to recover. There was no intervention in the CG, but in both IG and CG, the app carried out measurements (near-work distance, time, and illumination) in the background of the operating system and synchronized the data with a remote database using the wireless connection of the tablets. These were the only data that were synchronized (no personal details, photos, or other data were recorded). Due to the limited time we had for the measurements, it was decided to avoid a crossover design in which each child would be their own control.

The study was carried out over 15 days. In School #1, the total time the children used the app was 15 hours, on average 82 min per student. In School #2, the total time was 29 hours, on average 51 min per student. In total, over 10000 data samples were obtained.

## 3. Results

### 3.1. Face-Device Distance Measurements

**Figure 6:**
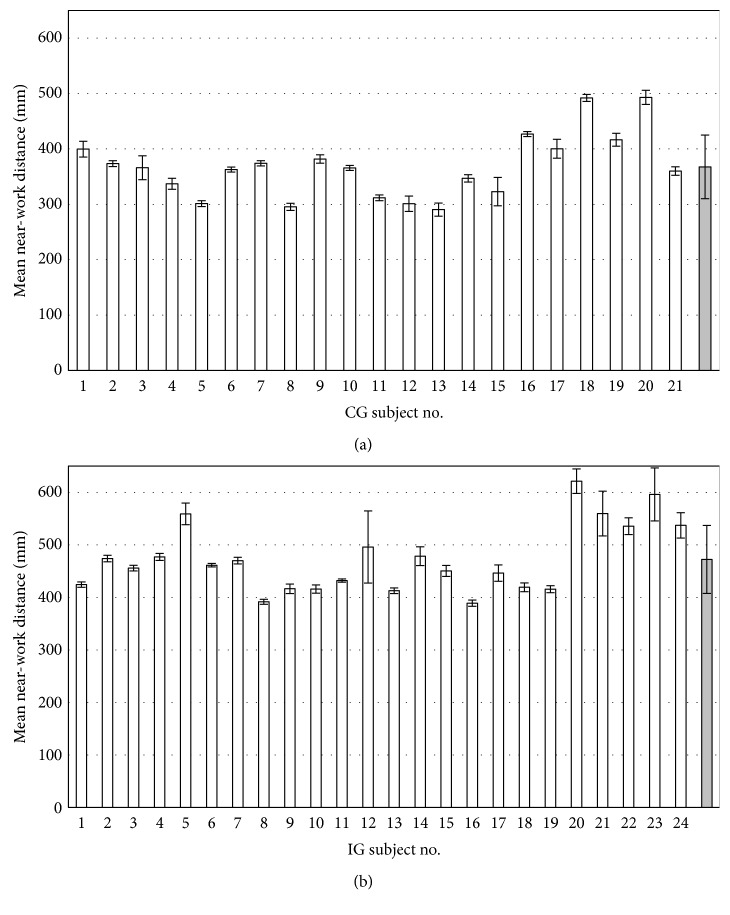
Mean (±1 SD) intersubject near-work distance of each child in the control group (CG) (a) and intervention group (IG) (b). The grey bars represent the mean for the entire group.

Figures 6(a) and 6(b) show the near-work distance measurements for each child in the CG and IG, respectively.

The mean face-device distance (grey bar) was 36.8 ± 5.7 cm in CG and 47.2 ± 6.5 cm in IG (grey bars on right of Figure 6), corresponding to 2.7 and 2.1 D of vergence, respectively. The percentage of children who used their devices at a distance greater than 40 cm was 24% and 92% in the CG and IG, respectively. When no distinction is made between children from different schools, the difference was statistically significant (*p*=9.85 10^−7^), just as when the same *t*-test was applied to the data from each school separately (*p* = 0.001 and 0.0001 for Schools #1 and #2, respectively). Habitual near-work distance did not differ between schools (*p*=0.56).

### 3.2. Illumination Measurements

**Figure 7:**
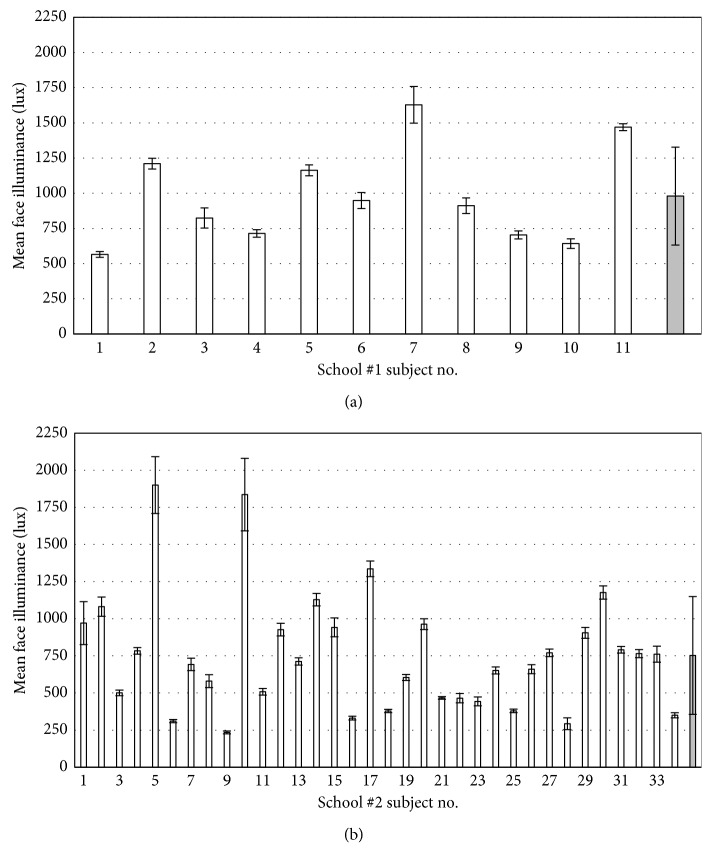
Mean (±1 SD) intersubject face illuminance in School #1 (a) and School#2 (b). The grey bars (last “subject”) represent the total mean for the entire group.

Figures 7(a) and 7(b) show the mean and SD face illuminance measurements for each child in School #1 and School #2, respectively.

The total mean classroom face illuminance (grey bar) was 980 ± 350 lux in School #1 and 750 ± 400 lux in School #2. The difference in face illuminance measurements between both Schools was significant (*p*=0.048).

## 4. Discussion

### 4.1. Face-Device Distance

Calibration revealed that devices equipped with the face-distance measuring app could measure viewing distance with an accuracy below 5 mm and a precision of approximately 1 cm or better (vergence error <0.03 D) within the range from 40 to 250 cm. This includes the typical mobile device use distance, which usually does not exceed one meter [11]. Additionally, it was found that the calibration performed at 200 cm resulted in slightly more accurate measurements compared to data collected after calibration at 60 cm.

Most students became familiar with the app very quickly, and after several times, the tablet's screen became dark, they learned to use and maintain it beyond the preconfigured distance of 40 cm (mean distance in the IG was 47.2 ± 6.5 cm). The results from CG indicate that the students' habitual face-device distance was 36.8 ± 5.7 cm when they performed near-work with their 10-inch tablets. There were no significant differences in habitual near-work distance in children from both schools (*p* > 0.05 between subjects in CG subdivided into both schools).

The total mean face-device distance in IG was 10.3 cm greater than in CG. The mean face-device distance in IG was greater than 40 cm for all subjects except for subjects #8 and #16, who were close (39.1 cm and 38.9 cm, respectively), which can be due to small individual calibration errors, forcing the darkness of the screen of the mobile device by under 40 cm. In dioptric terms, without taking into account errors of accommodation (or assuming that it was the same in both groups), this translated to a 0.6 D decrease in visual demand in IG compared to CG. In other words, the IG subjects had to accommodate less when using their devices. The precision with which the threshold for dimming was adapted to by the children indicate larger reductions in dioptric demand can be introduced by a simple adjustment to the app, e.g., set the preconfigured intervention distance to 50 cm and these students would have reduced their accommodative demand by approximately 1 D. The differences between mean intersubject near-work distances between IG and CG were significant (*p* < 0.05), with and without the Bonferroni correction [31], both when analyzing schools separately and together.

**Figure 8:**
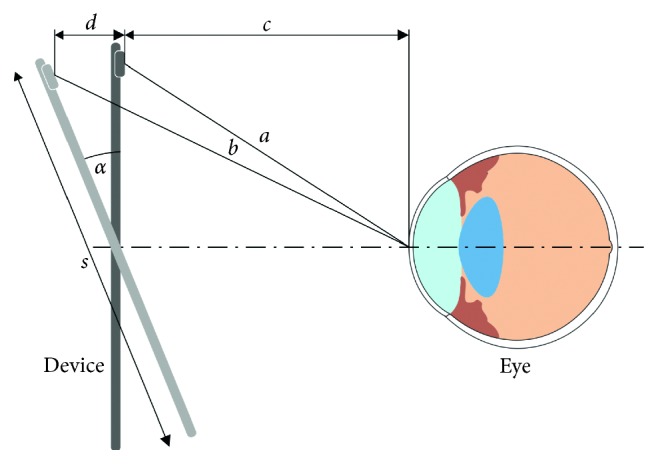
Change of distance from the camera C to the eye E of a tablet of height *s* when tilted by an angle *α* with respect to its original orientation parallel to the face.

The tilt of the device with respect to the face of the user could potentially give a wrong reading of the distance user-device.  Figure 8 shows a schematic of this limitation.

The error *b*—a of the measurement can be approximated by the formula in equation (1):

(3) $$\begin{array}{c} b-a\approx d=\frac{s}{2}\cdot \sin\;\alpha , \end{array}$$

where *d* is the approximate measurement error, *s* is the tablet height (*c* > *s*/2), and *α* is the tablet tilt.

In the present study, *s* = 25.4 cm and half of the field of view (FOV) of the camera of the device was 23°. Although the maximum error obtained from equation (1) is 5 cm, the face detection algorithm in the app used the whole height of the face, so the real maximum tilt angle is lower than 23°. Additionally, since the measurement error in equation (1) is linearly dependent on the tablet height, it can be expected to be lower when a smartphone is used instead.

### 4.2. Face Illuminance

**Figure 9:**
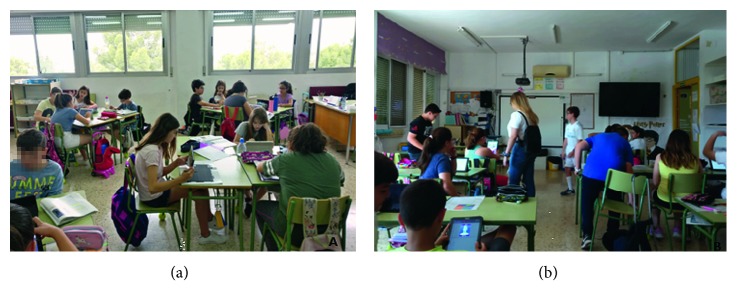
Use of tablets in two schools: School #1 (a) and School #2 (b).

Activities in School #1 were carried out with the blinds wide open and often with ceiling lights on. Meanwhile, in School #2, the majority of the blinds were rolled down and the ceiling lights were on (Figure 9). These observations were confirmed by the total mean values of face illuminance measured by tablets with the app: 980 ± 350 and 750 ± 400 lux in Schools #1 and #2, respectively (Figure 7). The measured illuminance levels were both higher than 300 lux required by law in the state of Murcia [32] and 652 lux reported by Read et al. as the average daily illuminance values which yielded low risk to increase myopia [33].

On the other hand, the measured mean illuminance values were 3 to 4 times higher than 248 ± 168 lux obtained in 2017 by Ostrin et al. using a wrist band device in school [25]. Since ambient light sensors included in smart watches and wrist bands are semiconductor devices similar to the ones included in smart phones and tablets, the higher measured illuminance with the tablets likely reflects the measurement geometry. A hand wearing a wrist band can often be lowered to the level of the hip or covered by clothes, whereas the tablets are always directed towards the face of the user and precalibrated in the lab to measure face illuminance. The app recorded data only when the child's face was present in the image from the front camera. The differences with previous studies can also be explained by the different geographical locations, season, and time of day. Both locations (Murcia and Houston) share similar latitudes (37 and 30 degrees North), but the former has a slightly larger annual solar exposure levels (2069 kWh/m^2^ compared to 1870 in Houston) [34].

The large variations in illumination between subjects (Figure 7) can be explained by the inhomogeneity in the illumination of the class in both schools. To test this hypothesis, the illuminance in different locations of the classroom in School #1 was measured using a lux meter at the same time of a sunny day. The difference between the minimum and maximum readings exceeded 600 lux. Direct exposure of the lux meter to sunlight was avoided (just like children would avoid direct sun in their face during a class). These values indicate that the illumination can vary as much as three times (possibly more) throughout the classroom.

During the measurement of illumination was being proceeded, we found a limitation which is the same as to the measure of distance. This restriction is on the tilt of the distance which can influence the measurement. In this particular case, it is highly important to bear in mind that the sensor must avoid a luminous focus above because it could cause the alteration of the measure, as a consequence. Thus, the most appropriate environment for this sensor is a room with a homogenous illumination due to the few changes, which could emerge in the measurement. The same would happen if whether we have a different skin tone, different clothing, or different background since it would not have as much impact as a bright focus above the sensor.

## 5. Conclusions

In summary, the results show that the proposed novel method of intervening with an app in excessive near work performed by schoolchildren while studying has accomplished its purpose of extending their habitual near-work distance without introducing any new elements into their environment. Thus, the novel method implemented in an app which was developed and tested in the present work can have potential impact on myopia onset and progression. By accurately measuring the near-work distance and face illuminance, the app has the ability to disentangle the independent contribution of each factor in myopia onset and progression. This needs to be tested in properly designed clinical trials, now made possible by the advances in the hardware of mobile devices.

It is also important to point out that there are many near work activities that children perform in the classroom that do not involve the use of an electronic devices (i.e., reading text books, or writing in their note books). For future research, other measures should be taken to control those activities carried out without the use of an electronic device.

## Data Availability

The data used in this study are available upon request to the corresponding author.
